# Biomarkers for Predicting the Occurrence and Progression of Atrial Fibrillation: Soluble Suppression of Tumorigenicity 2 Protein and Tissue Inhibitor of Matrix Metalloproteinase-1

**DOI:** 10.1155/2022/6926510

**Published:** 2022-12-31

**Authors:** Wei-Ping Sun, Xiao Du, Jun-Jun Chen

**Affiliations:** Department of Cardiology, Beijing Anzhen Hospital, Capital Medical University, Beijing Institute of Heart Lung and Blood Vessel Disease, Beijing, China

## Abstract

**Background:**

Soluble suppression of tumorigenicity 2 protein (sST2) and tissue inhibitor of matrix metalloproteinase (TIMP)-1 are involved in multiple pathogenic pathways, including cardiac remodeling, which is the main pathology of atrial fibrillation (AF). This study aims to investigate the previously unexplored relationship between the serum levels of sST2, TIMP-1, and AF.

**Methods:**

This was a prospective cross-sectional study conducted at the Capital Medical University Affiliated Beijing Anzhen Hospital between June 2019 and July 2020, with a total of 359 participants. The clinical characteristics and laboratory results of the patients were compared, and multivariable ordinal logistic regression was used to evaluate the relationship between serum sST2, TIMP-1, and AF.

**Results:**

The participants included 110 patients with sinus rhythm (SR), 113 with paroxysmal AF (the paroxysmal AF group), and 136 with persistent AF (the persistent AF group). It was found that the sST2 levels gradually increased in these three groups, from 9.1 (6.7–12.4 pg/ml) in the SR group to 14.0 (10.4–20.8 pg/ml) in the paroxysmal AF group and to 19.0 (13.1–27.8) pg/ml) in the persistent AF group (*p*  <  0.001). The multivariable ordinal logistic regression model for sST2 and TIMP-1 demonstrated that sST2 had an area under the receiver operating characteristic (ROC) curve (AUC) of 0.797 (95% confidence interval (CI) 0.749–0.846, *p*  <  0.001) and TIMP-1 had an AUC of 0.795 (95% CI 0.750–0.841, *p*=0.000). The multivariable ordinal logistic regression model for sST2 and TIMP-1 showed good discrimination between SR and AF, with an AUC of 0.846, and the addition of clinical factors, such as brain natriuretic peptide (BNP), left atrial diameter, age, and gender, to the biomarker model improved the detection of SR and AF (AUC 0.901).

**Conclusions:**

In this cohort study, sST2 and TIMP-1 were associated with AF progression, independent of clinical characteristics and biomarkers. Soluble ST2 and TIMP-1 combined with age, elevated N-terminal-pro hormone BNP(NT-BNP), and an enlarged left atrium were able to demonstrate the progression of AF reliably.

## 1. Introduction

Atrial fibrillation (AF) is the most common cardiac arrhythmia in clinical practice, and it is a major risk factor for stroke, heart failure (HF), and other cardiovascular-related complications [1]. In the current study, the prevalence of AF in adults was between 2% and 4%, and the annual rates of paroxysmal AF progression to persistent AF ranged from <1% to 15%, up to 27%–36% in studies with *a* ≥ 10-yearfollow-up [2, 3]. In other words, AF begets AF and is irreversible. The clinical outcome of patients with AF progression with regard to hospital admissions and major adverse cardiovascular events is worse compared with patients demonstrating no AF progression [4]. Catheter ablation is a widely accepted treatment for this type of arrhythmia, but the success rate is lower in patients with persistent AF than it is in those with paroxysmal AF [5].

There is increasing evidence that there is a link between oxidative processes and AF [6], and various inflammatory markers and mediators have been independently linked to AF, suggesting a strong association between inflammation and arrhythmia [7]. Inflammatory mediators can reflect changes in atrial electrophysiology and structural substrates, which lead to increased vulnerability to AF. The recent guidelines for AF diagnosis and treatment have shown that serum N-terminal-pro hormone brain natriuretic peptide (NT-proBNP) and left atrial diameters (LADs) are more powerful biomarkers than other clinical variables [1]. However, there is also ongoing research into novel biomarkers in the diagnosis and prognosis of AF. There are data indicating that C-reactive protein (CRP) and other proinflammatory cytokine levels are higher in blood samples drawn from patients with AF than those from patients with sinus rhythm (SR) [1], and the levels decrease gradually after catheter ablation of persistent or long-lasting AF [8]. In addition, soluble suppression of tumorigenicity 2 protein (sST2) has not been restricted to inflammation, but it is also expressed as a response to myocardial stress [9]. Tissue inhibitor of matrix metalloproteinase (TIMP)-1 is upregulated by inflammatory factors, such as interleukin [10], and AF progression is associated with a gradual increase in matrix metallopeptidase 9 (MMP-9)/TIMP-1 [11]. However, the role of these novel biomarkers in the progression of AF is not yet clear [11, 12], and neither is it clear whether serum sST2 and TIMP-1 can be detected and predict AF onset, especially the onset of paroxysmal AF. Clearly, an early diagnosis would be beneficial in the prevention of complications, such as HF and stroke.

This study aims to identify the clinical and laboratory variables that can predict the development of AF, ascertain whether the serum levels of sST2 and TIMP-1 are different between patients with SR and those with AF, observe whether sST2 and TIMP-1 are related to the progression of AF, and, if so, determine the cutoff point of sST2 for predicting such progression.

## 2. Methods

### 2.1. Study Design

In this cross-sectionalcase-control study, a review of the medical records of patients who were registered for catheter ablation to treat paroxysmal or persistent AF as well as the control group patients with SR was undertaken. The data collected included clinical variables, laboratory test results, and blood sample results. The study aimed to identify the clinical and laboratory variables differentiating the SR group, paroxysmal AF group, and persistent AF group and explore whether serum levels of sST2 and TIMP-1 could demonstrate AF progression.

### 2.2. Participants

A total of 249 inpatients with AF who were admitted to Capital Medical University Affiliated Beijing Anzhen Hospital between June 2019 and July 2020 were included in the study, along with 110 patients with SR, who constituted the control group. The inclusion criteria were as follows: patients aged >18 years, patients clinically diagnosed with AF, and patients who signed an informed consent form. The exclusion criteria were as follows: patients with a malignant tumor, inflammation, or other end-stage disease. The definition of paroxysmal AF used in this study was that AF terminates spontaneously or with intervention within seven days of onset, whereas persistent AF is continuously sustained beyond seven days, including episodes terminated by cardioversion (drugs or electrical cardioversion) after seven or more days. The diagnosis of paroxysmal or persistent AF was based on a 12-lead electrocardiogram and a 24-hour ambulatory electrocardiogram.

### 2.3. Data Collection

Clinical and laboratory data were extracted from the medical records by two independent doctors. The former included age, sex, and history of hypertension, coronary artery disease, diabetes, HF, and other similar indicators, and the laboratory data consisted of white and red blood cell counts, platelet counts, and hemoglobin (Hb) levels. In addition, the LAD, left ventricular end-systolic dimension, left ventricular end-diastolic dimension, and left ventricular ejection fraction were measured. Blood markers, namely, TIMP-1, sST2, high- sensitivity (hs)-CRP, and NT-proBNP were measured using the same blood sample within six hours of blood sampling. The blood sample was collected for paroxysmal and persistent patients during ongoing AF. The CHA2DS2-VASc and HAS-BLED scores, for the assessment of stroke and bleeding, respectively, were also calculated for all the participants.

### 2.4. Statistical Analysis

All statistical analyses were performed using SPSS 20.0 (IBM Corp., Armonk, NY, USA). Continuous data were expressed as mean ± standard deviation and analyzed using Student's *t*-test (for comparisons of two groups). Non-normally distributed continuous data were described as median and interquartile range, and comparisons between the groups were made using the Wilcoxon rank sum test. Categorical variables were presented as a number (percentage) and analyzed using the chi-square test or Fisher's exact test and considered appropriate. Parameters with *p*  <  0.05 in univariable ordinal logistic regression analysis were included in multivariate logistic regression analysis using the enter method. Multivariate ordinal logistic regression analysis was performed to assess the relationship between the serum levels of the biomarkers and the risk of AF, which was described using correlation coefficients and presented with 95% confidence intervals (CIs). To assess the discriminatory abilities of the biomarkers to predict AF, a receiver operating characteristic (ROC) curve was constructed, and the AUC was calculated. A value of *p*  <  0.05 was considered statistically significant.

## 3. Results

### 3.1. Baseline Characteristics

**Figure 1:**
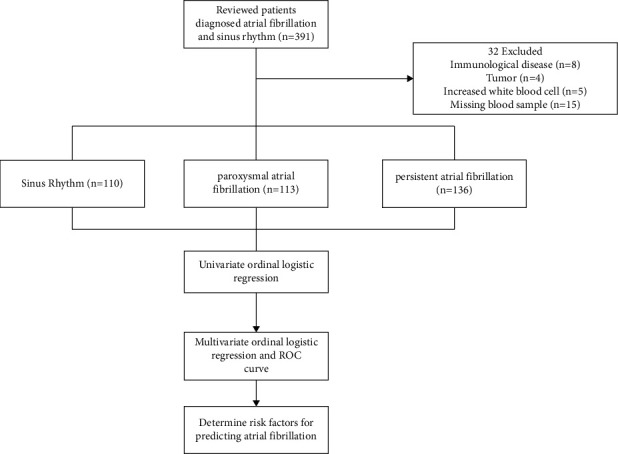
Patient selection flowchart.

A total of 359 participants were enrolled in the study between June 2019 and July 2020. Their clinical characteristics and laboratory data are summarized in Tables 1 and 2, respectively, and a patient selection flowchart is shown in Figure 1.  Table 1 shows that the age of patients with AF is higher than that of patients with SR, as follows: in the SR group 47 (36–62) years; in the paroxysmal AF group 62 (52–66) years; and in the persistent AF group 62 (52–69) years. The proportion of males in the AF groups is higher, as follows: SR group, 50 males (45.5%); paroxysmal AF group, 72 males (64.6%); and persistent AF group, 97 males (71.3%). The persistent AF group had a significantly higher body mass index (BMI) than the SR group (26.5 [24.2–29.1] vs. 25 [22.4–27.5], *p*=0.002), but there was little difference between the paroxysmal AF and SR groups (25.5 [23.5–28.4] vs. 25 [22.4–27.5], *p*=0.068) or the persistent AF and paroxysmal AF groups (26.5 [24.2–29.1] vs. 25.5 [23.5–28.4], *p*=0.258). Patients with AF tend to have hypertension, be smokers, and have a higher CHA2DS2-VASc score (Table 1). The echocardiography results also found that left atrial anteroposterior diameter is associated with the progression of AF.

The laboratory test data are shown in Table 2. Patients with AF had significantly higher levels of Hb than the SR group: the SR group, 144 (135–155) g/l; the persistent AF group, 152 (142–162.0) g/l, *p*  <  0.001; and the paroxysmal AF group, 148 (139–160) g/l, *p* = 0.012. The persistent AF group had the highest level of BNP among the three groups, and the difference was significant: the persistent AF group, 125 (87–215); the paroxysmal AF group, 67 (32–123); and the SR group, 32 (10–76.5), *p*  <  0.001). The serum level of hs-CRP was the highest in patients with persistent AF (1.2 [0.6–3.1] mg/l), and the difference between this group and the paroxysmal AF group (0.8 [0.5–1.9] mg/l) was significant (*p* = 0.022), as it was between the persistent AF group and the SR group (1 [0.45–1.9] mg/l, *p* = 0.0190. The serum levels of mean cell volume and mean cell hemoglobin were higher in the persistent AF group than those in the paroxysmal AF and SR groups. However, the serum levels of platelets, total protein, and albumin in the AF group were lower than those in in the SR group.

The serum levels of sST2 and TIMP-1 were significantly higher in both the paroxysmal AF and persistent AF groups than those in the SR group. The serum levels of sST2 increased significantly between the SR and the persistent AF group (SR group 9.1 [6.7–12.4], paroxysmal AF group 14 [10.4–20.8], *p*  <  0.001, and persistent AF group 19 [13.1–27.8], *p*  <  0.001). In addition, the serum level of TIMP-1 increased significantly from the SR group to the paroxysmal AF group and then to the persistent AF group as follows: SR group 67.4 (56.9–81.3) ng/ml; paroxysmal AF group 89.7 (70.6–115.5); and persistent AF group 127.3 (87.0–173.7), *p*  <  0.001. These results indicated that the serum levels of TIMP-1 and sST2 were associated with the progression of AF.

### 3.2. Multivariate Ordinal Logistic Regression Analysis of Factors Associated with Atrial Fibrillation

**Figure 2:**
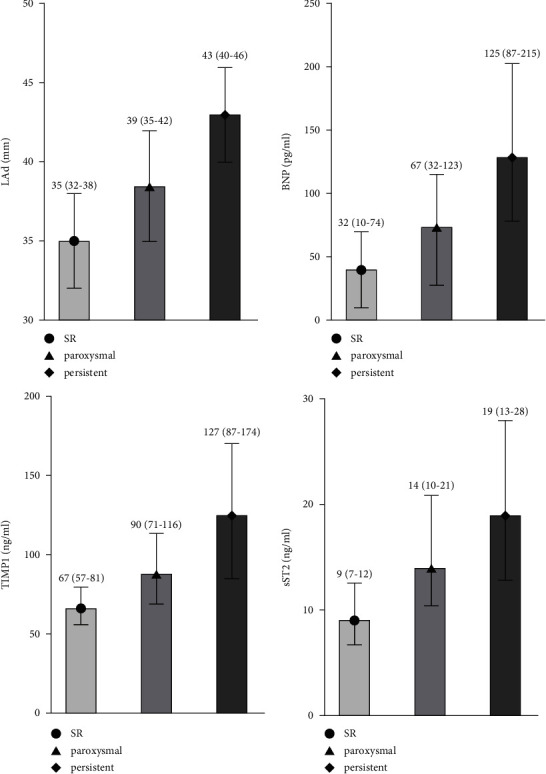
Soluble suppression of tumorigenicity 2 protein, tissue inhibitor of matrix metalloproteinase, and brain natriuretic peptide levels and the left atrial diameter.

The results of the univariate ordinal logistic regression analysis are shown in Table 3, which lists the variables that are independently associated with AF. All the independent risk factors associated with AF were included in the following multivariable ordinal logistic regression analysis. To identify the factors correlated with the progression of AF, a multivariable ordinal logistic regression analysis was performed. The results of the multivariable ordinal logistic regression analysis are detailed in Table 4. TIMP-1, sST2, BNP, and LAD were associated with the progression of AF as their levels gradually increased starting from the SR group to the paroxysmal AF group and then to the persistent AF group (Figure 2).

**Figure 3:**
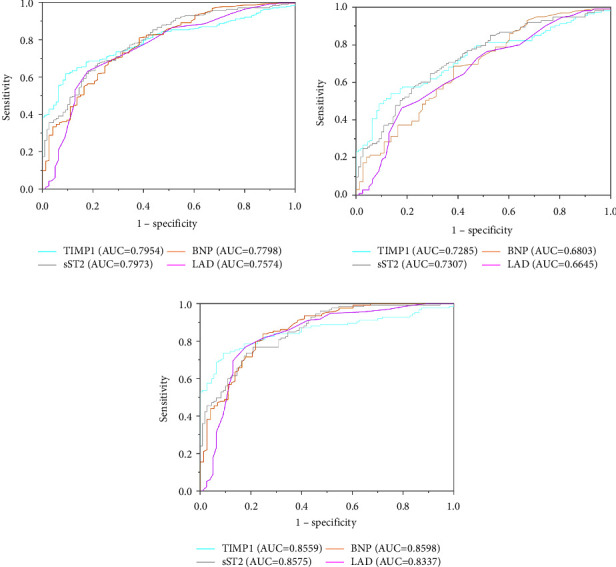
A receiver operator characteristic curve analysis for the progression of atrial fibrillation. (a) Sinus rhythm and atrial fibrillation. (b) Sinus rhythm and paroxysmal atrial fibrillation. (c) Sinus rhythm and persistent atrial fibrillation.

**Figure 4:**
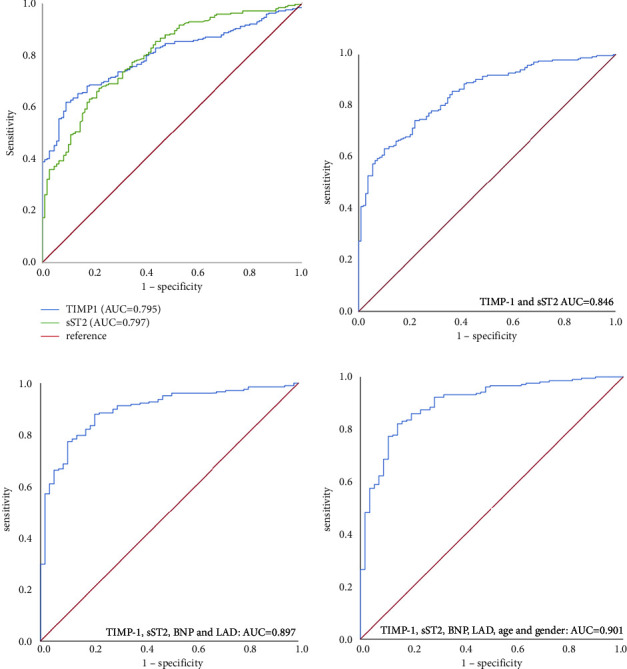
A receiver operator characteristics curve analysis with different factors for assessing the progression of atrial fibrillation. (a) Tissue inhibitor of matrix metalloproteinase (TIMP-1) and soluble suppression of tumorigenicity 2 protein (sST2) individually. (b) TIMP-1 and sST2 combined. (c) TIMP-1, sST2, brain natriuretic peptide (BNP), and left atrial diameter (LAD) combined. (d) TIMP-1, sST2, BNP, LAD, age, and gender combined.

The ROC curve for assessing the ability of these factors to predict AF progression is shown in Figures 3 and 4. The ROC curve shows that sST2 and TIMP-1 were the best biomarkers for predicting the progression of AF (Figure 3). The AUC of sST2 for the progression of AF was 0.7973 (95% CI 0.749–0.8456; *p*  <  0.001). According to the highest Youden's index, the cutoff value with the highest sensitivity and specificity was 12.81 ng/ml of sST2 (78.4% and 75.4%, respectively). The AUC for predicting AF (both paroxysmal and persistent AF) was 0.7954 (95% CI 0.813–0.922, *p*  <  0.001). According to the highest Youden index, the cutoff of TIMP-1 for predicting AF was 90.57 ng/ml, and sensitivity and specificity were 63% and 94.7%, respectively. Both sST2 and TIMP-1 had a high predictive value for the progression of AF. However, although the serum level of sST2 had both high sensitivity and specificity, the prediction value of TIMP-1 was even higher. Thus, a combination of sST2 and TIMP-1 could effectively predict the progression of AF.

The ROC curve for assessing the ability of sST2 and TIMP-1 alone and in combination with other biomarkers and clinical parameters to predict AF progression is shown in Figure 4. When these two biomarkers were combined, the AUC was 84.6% (95% CI 0.806–0.887, *p*=0.000), sensitivity was 63.4%, and the specificity was 90.0%. When these two biomarkers were combined with BNP and LAD, the AUC was 89.7% (95% CI 0.855–0.939, *p*=0.000) and the sensitivity and specificity were 88.0% and 78.9%, respectively. Finally, the combination of the two biomarkers with BNP and LAD as well as age and gender resulted in an AUC of 0.901 (95% CI 0.858–0.944, *p*=0.000). The sensitivity and specificity of these six variables for predicting AF progression were 82.2% and 86.0%, respectively. The clinical models of TIMP-1 and sST2 for predicting AF progression showed a modest improvement with the addition of BNP, LAD, age, and gender.

## 4. Discussion

There are two main findings in this present study. The serum level of sST2 and TIMP-1 may be a biomarker for differentiating paroxysmal AF from SR when AF cannot be diagnosed using electrocardiography in clinical practice. The cutoff values of TIMP and sST2 were 90.57 ng/ml and 12.81 ng/ml, respectively, and both of them had high sensitivity and specificity. TIMP-1 and sST2 may be AF treatment targets.

The lifetime risk of AF mainly depends on age, but it is also influenced by genetic and clinical factors (including gender, BMI, smoking history, HF, and hypertension) [1]. This study found that age and BMI are higher in the AF group, as is the proportion of males, which is consistent with one previous study [13], and the AF group also had a wider LAD, as found elsewhere [14]. Although the early intervention and control of modifiable risk factors were previously found to reduce the incidence of AF [13], the predictive value of biomarkers has not been well-defined until now.

Atrium enlargement can result in physiological stretching in AF. Physiological stretching causes myofibroblasts to release IL-33, which binds the ST2 receptor (ST2L) to the cardiomyocyte membrane, promoting cell survival and integrity. In chronic conditions, however, local and neighboring cells can increase the release of the IL-33 decoy, sST2, which blocks IL-33/ST2L binding and promotes tissue fibrosis. Novel aspects of ST2/IL-33 signaling mediating cardiac fibrosis represent some new biomolecular targets for the prevention and treatment of maladaptive remodeling and disease progression [15]. Matilla et al. found that sST2 could affect myofibroblast activation, leading to an increase in collagen synthesis and profibrotic molecules in human cardiac fibroblasts. NRP-1, a molecule upregulated by Sst2, has emerged as an interesting new target in cardiac fibrosis. The proinflammatory and profibrotic effects triggered by sST2 via nuclear factor kappa-light-chain-enhancer of activated B cells highlighted the key role of the latter on cardiac fibrosis [16]. In this cross-sectional study, the role of the biomarkers sST2 and TIMP-1 in predicting the progression of AF was explored. Previous studies had led to a number of findings. The systemic biomarker sST2 was independently associated with increased primary outcomes in patients with HF and AF, and the prognostic performance of sST2 was stronger in AF for all-cause mortality [17]. Higher sST2 was associated with a higher prevalence of AF, possibly reflecting remodeling phenomena in AF [18], and, in patients with persistent AF, increased sST2 served as a marker of recurrence after radiofrequency ablation, and patients with sST2 ≥ 39.25 ng/ml were more likely to have a recurrence within one year [19]. In the current study, the results also showed a higher serum level of sST2 in patients with persistent AF. In another study, patients with AF showed significantly higher sST2 than the control group did [20]. This study consistently found that sST2 was higher in patients with SR and increased gradually as a patient moved from SR to paroxysmal persistent AF.

Atrial fibrillation is dependent on the electrical and structural remodeling of the atrium, and myocardial fibrosis plays a critical role in the maintenance of AF through the heterogeneity of atrial electrical conduction [21]. Atrial structural remodeling and maintenance depend on the synthesis and degradation of extracellular matrix (ECM) proteins (matrix metalloproteinases [MMPs] and TIMPs) [21]. An imbalance between MMPs and TIMPs can lead to an abnormal turnover of the ECM and result in atrial remodeling and fibrosis. In 15 rapid atrial pacing-induced AF pig models, the MMPs and TIMPs of in situ activity and the expression of gelatinases (MMP-2 and MMP-9) and their relationship with TIMP-1 in the atria were explored. Chen et al. found that in situ gelatinase activity was significantly higher in AF than in SR. The significant increases in MMP-9 in its pro-form and the messenger ribonucleic acid level were shown to be responsible for the increased gelatinase activity in AF. The inhibitory activities of glycosylated TIMP-1 and TIMP-3 in AF tissues were markedly elevated and localized in the atrial interstitium. In addition, although TIMP-1 was found to be mostly colocalized with gelatinase activity in the AF tissues, implying the coexistence of gelatinase activity and TIMP-1, TIMP-3 appeared only partially colocalized and halted the gelatinase activity surrounding the cardiomyocytes. The increased activity of gelatinase, TIMP-1, and TIMP-3 as well as their interaction may have contributed to the atrial ECM remodeling of AF [22]. In other studies, in patients with persistent AF, TIMP-1 levels were increased when compared with patients with paroxysmal AF, and the levels of TIMP-1 were also higher in patients with paroxysmal AF than in the SR group [23, 24]. These findings were consistent with the present study. In another recent study, the expression of TIMP-1 was high in patients with persistent AF and chronic AF but not in those with paroxysmal AF [25], and elsewhere it was found that there was no significant difference in the plasma levels of TIMP-1 in patients with paroxysmal AF or those with persistent AF [24], which differed slightly from the findings of the present study. The reason may be due to inadequate power (i.e., a type II error). In the present study, TIMP-1 was higher in the paroxysmal AF group than the SR group and higher in the persistent AF group than the paroxysmal AF group. In one study, AF development and progression (from paroxysmal to persistent) were associated with a gradual increase in the serum levels of TIMP-1 [11]. A recent meta-analysis revealed that increased MMP-1 and decreased TIMP-2 levels are significantly associated with an increased risk of AF [26]. Moreover, Wakula et al. demonstrated that the levels of TIMP-1 in patients with paroxysmal AF were higher than those in patients without AF [27].

The clinical course of AF is marked by the development of atrial fibrosis [28–31], and sST2 is a marker of fibrosis, particularly cardiac fibrosis [15]. Elevated sST2 levels in patients with AF were associated with higher left atrium low-voltage areas [32]. Moreover, sST2 levels were higher in paroxysmal AF with a low-voltage zone greater than 20% compared to those with a smaller low-voltage zone. The dynamic balance of MMP-9 and TIMP-1 determines the fibrosis signal strength [21], and immunohistochemical and double-immunofluorescence staining for TIMP-1 in AF and SR tissues in the atrial interstitium showed that the increased levels of TIMP-1staining in the atrial interstitium reached statistical significance in the AF group when compared with the SR group [22]. Therefore, these two biomarkers have high specificity for left atrial remodeling.

Brain natriuretic peptide is an indicator of cardiac wall stress, and it is responsible for fluid and blood pressure homeostasis through its diuretic and vasodilatory effects and also affects cardiovascular remodeling. Thus, there is a strong causal relationship between natriuretic peptides and the incidence of AF [33, 34]. Moreover, the most prominent risk factors for AF development, such as age, sex, increased BMI, hypertension, and HF, have all been related to elevated natriuretic peptide concentrations. Previous studies showed that patients with AF had significantly higher CRP levels than the SR group [20], and elevated hs-CRP levels were significantly associated with an increased risk of AF [35–37]. However, circulating BNP and CRP are known biomarkers for systemic circulation and can be influenced by many factors, such as HF and acute or chronic inflammation. In the present study, the serum levels of sST2 and TIMP-1 played an important role in AF progression. These two biomarkers had high sensitivity and specificity for differentiating SR from AF, especially when combined with BNP, LAD, age, and gender. Compared to the clinical variables, sST2 and TIMP-1 had high sensitivity and specificity for differentiating SR and AF, as they were involved in the development of AF, but BNP did not. The increased sST2 levels suggested that the processes of inflammation and fibrosis were overactivated. However, unlike BNP, the expression of sST2 was not influenced by age or BMI.

## 5. Limitations

This study had some limitations. First, it was an observational study with a relatively small sample size in each group. Certain known predictors of recurrence, such as hs-CRP, were not found to be significant in this study, which may have been because of a lack of statistical power. Therefore, research with a larger population is necessary to confirm the prognostic value of sST2 and TIMP-1 in identifying patients at high risk of paroxysmal AF. Second, serum markers are not heart-specific, and the findings here were not supported with atrial tissue biopsy data or coronary sinus sampling. Moreover, patients with conditions associated with fibrosis were excluded from the study. In addition, the fact that this was a prospective, single-center, and cross-sectional study may have introduced unavoidable selection bias. Thus, to further confirm the efficiency of sST2 and TIMP-1 in evaluating AF progression, a prospective and multicenter study is required. Finally, the relationship between sST2, TIMP-1, and atrial tissue fibrosis was not investigated in this study, and this issue needs to be addressed.

## 6. Conclusions

In patients with AF, serum TIMP-1, sST2, BNP, hs-CRP, and LAD are associated with the progression of AF. In this study, sST2 and TIMP-1, which both had high sensitivity and specificity, were the best biomarkers for evaluating the progression of AF. Therefore, in clinical practice, sST2 and TIMP-1 may be able to serve as biomarkers to differentiate paroxysmal AF from SR.

## Figures and Tables

**Table 1:** Baseline clinical characteristics.

Characteristic	Sinus rhythm (*n* = 110)	Paroxysmal AF (*n* = 113)	Pa value	Persistent AF (*n* = 136)	Pb value	Pc value
Age (years)	47.0 (36.0–62.0)	62.0 (52.0–66.0)	<0.001	62.0 (52.0–69.0)	<0.001	0.436
Male (*n*, %)	50 (45.5)	72 (63.7)	0.006	97 (71.3)	<0.001	0.201
BMI	25.0 (22.4–27.5)	25.5 (23.5–28.4)	0.060	26.5 (24.2–29.1)	0.003	0.237
CAD	5 (4.6)	13 (11.5)	0.056	16 (11.8)	0.044	0.949
HTN	28 (25.5)	57 (50.4)	<0.001	78 (57.4)	<0.001	0.276
DM	13 (11.8)	16 (14.2)	0.603	17 (12.5)	0.871	0.701
HF	4 (3.6)	4 (3.6)	0.979	12 (8.8)	0.101	0.094
Smoking	9 (8.2)	38 (33.6)	<0.001	28 (20.6)	0.007	0.020
Drinking	4 (3.6)	4 (3.5)	0.969	11 (8.1)	0.147	0.133
CHA2DS2-VASc (*n*, %)			0.034		0.007	0.629
0 or 1	81 (73.6)	66 (58.4)		74 (54.4)		
2 or 3	25 (22.7)	36 (31.9)		51 (37.5)		
≥4	4 (3.6)	11 (9.7)		11 (8.1)		
Mean	0.45	1.5	0.04	1.69	0.004	0.378
HAS-BLED score						
≥3 (*n* (%))	2 (1.8)	8 (7.1)	0.058	5 (3.7)	0.383	0.229
LA (mm)	35.0 (32.0–38.0)	38.5 (35.0–42.0)	<0.001	43.0 (40.0–46.0)	<0.001	<0.001
LVEF (%)	65.0 (60.0–68.0)	63.0 (60.0–66.0)	0.031	61.0 (56.0–66.0)	<0.001	0.015
LVEDD (mm)	47.5 (45.0–50.0)	47.0 (45.0–50.0)	0.387	48.5 (45.0–52.0)	0.112	0.005
LVESD (mm)	30.0 (28.0–33.0)	31.0 (28.0–33.0)	0.555	32.0 (28.0–36.0)	0.032	0.046

CAD: coronary artery disease; HTN: hypertension; DM: diabetes mellitus; HF: heart failure; ACEI: angiotensin-converting enzyme inhibitor; ARB: angiotensin receptor blocker; CCB: calcium channel blocker; LA: left atrium diameter; LVEF: left ventricular ejection fraction; LVEDD: left ventricular end-diastolic dimension; LVESD: left ventricular end-systolic dimension. Pa: paroxysmal vs. control, Pb: persistent vs. control, and Pc: persistent vs. paroxysmal.

**Table 2:** Laboratory examination of participants stratified according to AF status.

Characteristic	Sinus rhythm (*n* = 110)	Paroxysmal AF(*n* = 113)	Pa value	Persistent AF(*n* = 136)	Pb value	Pc value
RBC (10^9^/l)	4.7 (4.4–5.0)	4.7 (4.4–5.0)	0.712	4.8 (4.5–5.2)	0.014	0.033
WBC (10^9^/l)	6.5 (5.6–7.7)	6.4 (5.7–7.6)	0.872	6.6 (5.5–7.5)	0.844	0.827
PLT (10^12^/l)	237.5 (206.0–281.0)	215.0 (184.0–246.0)	0.001	210.0 (178.5–243.5)	<0.001	0.393
Hb (g/l)	144.0 (135.0–155.0)	148.0 (139–160)	0.012	152.0 (142.0–162.0)	<0.001	0.155
CREA (umol/l)	62.1 (52.4–75.2)	69.9 (59.8–80.2)	0.001	74.0 (63.6–82.9)	<0.001	0.116
GLU (mmol/l)	5.2 (4.9–5.8)	5.5 (5.1–6.1)	0.004	5.5 (5.1–6.6)	<0.001	0.190
GA (%)	13.4 (12.4–14.8)	14.1 (13.3–15.3)	0.013	14.3 (13.0–15.9)	0.008	0.651
HCY (umol/l)	11.8 (9.2–14.1)	12.6 (10.7–15.2)	0.059	13.7 (11.7–17.8)	<0.001	0.015
TIMP-1 (ng/ml)	67.4 (56.9–81.3)	89.7 (70.6–115.5)	<0.001	127.3 (87.0–173.7)	<0.001	<0.001
sST2 (pg/ml)	9.1 (6.7–12.4)	14.0 (10.4–20.8)	<0.001	19.0 (13.1–27.8)	<0.001	<0.001
BNP (pg/ml)	32.0 (10.0–74.0)	67.0 (32.0–123.0)	<0.001	125.0 (87.0–215.0)	<0.001	<0.001
ALT (U/l)	18.5 (14.0–29.0)	21.0 (16.0–29.0)	0.222	21.0 (15.5–29.0)	0.303	0.766
AST (U/l)	21.5 (18.0–26.0)	22.0 (18.0–26.0)	0.549	22.0 (18.0–28.0)	0.253	0.585
GGT	23.0 (16.0–40.0)	26.0 (18.0–37.0)	0.272	30.5 (22.0–39.0)	0.009	0.122
TP (g/l)	74.9 (70.8–77.4)	71.4 (67.0–75.4)	<0.001	72.7 (68.8–75.8)	0.003	0.189
Alb (g/l)	47.0 (44.1–49.4)	45.3 (42.5–47.8)	0.002	44.9 (42.6–47.3)	<0.001	0.513
Glo (g/l)	27.6 (24.1–30.1)	26.2 (23.1–28.7)	0.026	27.3 (25.0–30.2)	0.772	0.010
Tbil (umol/l)	10.9 (8.1–14.6)	12.8 (9.9–16.8)	0.001	14.3 (10.9–20.0)	<0.001	0.029
Dbil (umol/l)	3.0 (2.3–4.0)	3.5 (2.5–4.5)	0.099	4.4 (3.2–6.3)	<0.001	<0.001
Ibil (umol/l)	8.0 (4.8–10.9)	10.0 (6.9–12.8)	0.001	10.3 (6.8–14.0)	<0.001	0.532
LDH (U/l)	183 (160–208.0)	183.0 (159.0–208.0)	0.810	194.0 (174.5–219.5)	0.012	0.004
TG (mmol/l)	1.3 (0.9–2.0)	1.5 (1.0–2.3)	0.103	1.2 (0.9–1.8)	0.800	0.031
Tcho (mmol/l)	4.7 (3.9–5.5)	4.4 (3.9–5.2)	0.176	4.2 (3.7–4.9)	0.001	0.048
sdLDLc	0.7 (0.5–1.0)	0.8 (0.5–1.1)	0.207	0.7 (0.4–1.1)	0.510	0.057
LDL-c (mmol/l)	3.0 (2.3–3.5)	2.7 (2.1–3.4)	0.415	2.4 (1.7–3.1)	0.002	0.022
HDL-c (mmol/l)	1.2 (1.1–1.5)	1.2 (1.0–1.4)	0.001	1.2 (1.0–1.4)	0.089	0.109
hs-CRP (mg/l)	1.0 (0.5–1.9)	0.8 (0.5–1.9)	0.871	1.2 (0.6–3.1)	0.019	0.022
D-dimer (ng/ml)	83.0 (52.0–120.0)	73.0 (44.0–106.0)	0.187	71 (40.0–133.0)	0.408	0.807
FDP (ug/ml)	0.4 (0.1–0.7)	0.4 (0–0.7)	0.453	0.3 (0–0.8)	0.939	0.583
FIB (g/l)	2.9 (2.5–3.3)	3.0 (2.5–3.4)	0.391	3 (2.7–3.4)	0.105	0.527
HCT (%)	42.1 (39.4–44.6)	42.1 (40.5–44.7)	0.379	43.4 (41.3–46.0)	0.001	0.011
MCV (fl)	88.6 (86.2–90.7)	90.3 (88.1–92.5)	0.001	90.5 (88.1–92.9)	0.001	0.752
MCH (pg)	30.5 (29.8–31.7)	31.7 (30.7–32.8)	<0.001	31.3 (30.5–32.6)	<0.001	0.162
MCHC (g/l)	342.5 (334.0–352.0)	351.0 (341.0–360.0)	<0.001	344.0 (337.5–356.0)	0.186	0.017
RDW-SD (fl)	41.8 (40.4–43.5)	42.3 (41.0–44.4)	0.065	42.7 (41.4–45.1)	0.004	0.245
RDW-CV (%)	13.0 (12.6–13.4)	13.0 (12.7–13.4)	0.811	13.1 (12.7–13.6)	0.145	0.222
MPV (fl)	10.7 (10.0–11.4)	10.6 (10.1–11.3)	0.930	10.7 (10.0–11.1)	0.378	0.489
PCT (%)	0.3 (0.2–0.3)	0.2 (0.2–0.3)	0.001	0.2 (0.2–0.3)	<0.001	0.333
PDW (%)	13.2 (12.0–15.2)	12.5 (11.6–14.3)	0.043	13.3 (11.8–15.4)	0.898	0.032
PLCR	30.2 (24.8–36.6)	29.4 (25.4–35.5)	0.665	29.8 (24.9–34.1)	0.276	0.619

WBC: white blood cell; RBC: red blood cell; PLT: platelet count; Hb: hemoglobin; UA: uric acid; CREA: creatinine; Glu: fasting blood glucose; GA: glycated albumin; HCY: homocysteine; TIMP-1: tissue inhibitors of metalloproteinase-1; sST2: soluble suppression of tumorigenicity 2; BNP: B-type natriuretic peptide; ALT: alanine aminotransferase; AST: aspartate transaminase; TP: total protein; Alb: albumin; Glo: globulin; Tbil: total bilirubin; Dbil: direct bilirubin; Ibil: indirect bilirubin; LDH: lactate dehydrogenase; TG: triacylglycerol; Tcho: total cholesterol; LDL-c: low-density lipoprotein cholesterol; HDL-c: high density lipoprotein cholesterol; LP(a): lipoprotein(a); FFA: free fatty acid; C1q: anti-complement 1q antibody; hs-CRP: high-sensitivityC-reactive protein; FDP: fibrinogen degradation products; FIB: fibrinogen; HCT: hematocrit; MCV: mean red blood cell volume; MCH: mean corpuscular hemoglobin; MCHC: mean corpuscular hemoglobin concentration; RDW-SD: red blood cell distribution width-standard deviation; RDW-CV: red blood cell distribution width-cell volume; MPV: mean platelet volume; PCT: platelet hematocrit; PDW: platelet distribution width; Pa: paroxysmal vs. control, Pb: persistent vs. control, and Pc: persistent vs. paroxysmal.

**Table 3:** Univariate ordinal logistic regression analysis of the factors with sinus rhythm and paroxysmal, persistent AF.

Variable	Coef	SE	95% CI	*p* value
Age (years)	0.055	0.008	0.039–0.071	<0.001
Male	0.827	0.204	0.427–1.226	<0.001
BMI	0.059	0.023	0.013–0.105	0.011
HTN *n* (%)	0.968	0.201	0.575–1.361	<0.001
CHA2DS2-VASc	0.214	0.075	0.068–0.360	0.004
HAS-BLED	0.502	0.116	0.274–0.731	<0.001
LAd (mm)	0.160	0.021	0.119–0.201	<0.001
LVEF (%)	−0.054	0.016	−0.085 to −0.023	0.001
LVESD (mm)	0.059	0.021	0.018–0.100	0.005
PLT (10^12^/l)	−0.007	0.002	−0.011 to −0.004	<0.001
Hb (g/l)	0.019	0.006	0.006–0.031	0.003
CREA (umol/l)	0.025	0.006	0.013–0.037	<0.001
HCY (umol/l)	0.029	0.013	0.004–0.053	0.022
TIMP-1 (ng/ml)	0.024	0.003	0.019–0.030	<0.001
sST2 (pg/ml)	0.126	0.015	0.096–0.155	<0.001
BNP (pg/ml)	0.011	0.002	0.008–0.014	<0.001
TP (g/l)	−0.046	0.018	−0.081 to −0.011	0.010
Alb (g/l)	−0.095	0.025	−0.144 to −0.045	<0.001
Tbil (umol/l)	0.082	0.017	0.048–0.115	<0.001
Dbil (umol/l)	0.317	0.055	0.210–0.424	<0.001
Ibil (umol/l)	0.072	0.020	0.034–0.111	<0.001
LDH (U/l)	0.005	0.002	0.001–0.009	0.007
Tcho (mmol/l)	−0.325	0.096	−0.513 to −0.137	0.001
LDL-c (mmol/l)	−0.217	0.096	−0.405 to −0.030	0.023
hs-CRP (mg/l)	0.085	0.027	0.031–0.138	0.002
FDP (ug/ml)	0.199	0.094	0.014–0.383	0.035
HCT (%)	0.085	0.025	0.035–0.135	0.001
MCV (fl)	0.062	0.022	0.020–0.105	0.004
MCH (pg)	0.112	0.054	0.007–0.218	0.037
RDW-SD (fl)	0.066	0.035	−0.003 to 0.135	0.062

BMI: body mass index; HTN: hypertension; LAd: left atrium diameters; LVEF, left ventricular ejection fraction; LVESD, left ventricular end of systolic dimeter; PLT, platelet count; Hb, hemoglobin; CREA: creatinine; HCY: homocysteine; TIMP-1: tissue inhibitors of metalloproteinase-1; sST2: soluble suppression of tumorigenicity 2; BNP: B-type natriuretic peptide; TP: total protein; Alb: albumin; Glo: globulin; Tbil: total bilirubin; Dbil: direct bilirubin; Ibil: indirect bilirubin; LDH: lactate dehydrogenase; Tcho: total cholesterol; LDL-c: low-density lipoprotein cholesterol; hs-CRP: high-sensitivityC-reactive protein; FDP: fibrinogen degradation products; HCT: hematocrit; MCV: mean red blood cell volume; MCH: mean corpuscular hemoglobin; RDW-SD: red blood cell distribution width-standard deviation.

**Table 4:** Multivariable predictors of atrial fibrillation progression among patients in the cohort.

Variates	Coef (95% CI)	OR	95% CI	*p* value
Age (years)	0.020 (−0.002, 0.041)	1.020	0.998–1.042	0.069
Gender (M, %)	0.435 (−0.124,0.994)	1.544	0.883–2.701	0.128
LAd (mm)	0.089 (0.036, 0.143)	1.094	1.006–1.019	0.001
TIMP-1 (ng/ml)	0.013 (0.006, 0.019)	1.013	1.006–1.019	<0.001
sST2 (pg/ml)	0.082 (0.045, 0.120)	1.086	1.046–1.272	<0.001
BNP (pg/ml)	0.005 (0.002, 0.009)	1.005	1.002–1.009	0.002

## Data Availability

The data that support the findings of this study are available from the corresponding author upon reasonable request.
